# Energy Cost of Force Production After a Stretch-Shortening Cycle in Skinned Muscle Fibers: Does Muscle Efficiency Increase?

**DOI:** 10.3389/fphys.2020.567538

**Published:** 2021-01-18

**Authors:** Venus Joumaa, Atsuki Fukutani, Walter Herzog

**Affiliations:** ^1^Human Performance Laboratory, Faculty of Kinesiology, University of Calgary, Calgary, AB, Canada; ^2^Faculty of Sport and Health Science, Ritsumeikan University, Kusatsu, Japan; ^3^Biomechanics Laboratory, School of Sports, Federal University of Santa Catarina, Florianopolis, Brazil

**Keywords:** mechanical work, ATPase activity, cross-bridge cycling, stiffness, force depression, residual force enhancement, titin

## Abstract

Muscle force is enhanced during shortening when shortening is preceded by an active stretch. This phenomenon is known as the stretch-shortening cycle (SSC) effect. For some stretch-shortening conditions this increase in force during shortening is maintained following SSCs when compared to the force following a pure shortening contraction. It has been suggested that the residual force enhancement property of muscles, which comes into play during the stretch phase of SSCs may contribute to the force increase after SSCs. Knowing that residual force enhancement is associated with a substantial reduction in metabolic energy per unit of force, it seems reasonable to assume that the metabolic energy cost per unit of force is also reduced following a SSC. The purpose of this study was to determine the energy cost per unit of force at steady-state following SSCs and compare it to the corresponding energy cost following pure shortening contractions of identical speed and magnitude. We hypothesized that the energy cost per unit of muscle force is reduced following SSCs compared to the pure shortening contractions. For the SSC tests, rabbit psoas fibers (*n* = 12) were set at an average sarcomere length (SL) of 2.4 μm, activated, actively stretched to a SL of 3.2 μm, and shortened to a SL of 2.6 or 3.0 μm. For the pure shortening contractions, the same fibers were activated at a SL of 3.2 μm and actively shortened to a SL of 2.6 or 3.0 μm. The amount of ATP consumed was measured over a 40 s steady-state total isometric force following either the SSCs or the pure active shortening contractions. Fiber stiffness was determined in an additional set of 12 fibers, at steady-state for both experimental conditions. Total force, ATP consumption, and stiffness were greater following SSCs compared to the pure shortening contractions, but ATP consumption per unit of force was the same between conditions. These results suggest that the increase in total force observed following SSCs was achieved with an increase in the proportion of attached cross-bridges and titin stiffness. We conclude that muscle efficiency is not enhanced at steady-state following SSCs.

## Introduction

Muscle force is enhanced during shortening, when shortening is preceded by an active stretch. This phenomenon is known as the stretch-shortening cycle (SSC) effect. The detailed molecular mechanisms responsible for the increase in force and work during SSCs remain unknown. It has been suggested that the SSC effect may be caused by tendon elongation (Cavagna and Citterio, 1974; Finni et al., 2001; Kawakami et al., 2002), stretch-induced reflex responses (Nichols and Houk, 1973; Dietz et al., 1979), pre-activation (cross-bridge elongation) of muscles (Bobbert et al., 1996; Bobbert and Casius, 2005), and residual force enhancement (Seiberl et al., 2015; Fukutani et al., 2017).

Recently, in experiments performed in skinned muscle fibers, Fukutani et al. (2017) showed that the increase in force observed in the shortening phase of SSCs can be maintained at steady-state following stretch-shortening compared to pure shortening contractions of identical speed and magnitude. Fukutani et al. (2017) argued that since experiments were performed in skinned muscle fibers, in which the effects of tendon elongation and reflex activation were eliminated, and the effects of muscle pre-activation (associated with cross-bridge elongations) dissipate quickly, residual force enhancement is likely the primary factor causing the increase in steady-state force following SSCs. This hypothesis was further supported by studies on *in vivo* muscles (Seiberl et al., 2015; Fortuna et al., 2019).

Abbott and Aubert (1952) were the first to systematically show that the steady-state isometric force after active stretching of muscles was higher than the purely isometric force at the corresponding lengths. This property of skeletal muscle became known as residual force enhancement and has been systematically observed in whole muscle preparations (Morgan et al., 2000; Herzog and Leonard, 2002; Pinniger et al., 2006), human skeletal muscles (Oskouei and Herzog, 2006; Shim and Garner, 2012; Power et al., 2013), single fibers (Sugi and Tsuchiya, 1988; Edman and Tsuchiya, 1996; Pinnell et al., 2019), and single myofibrils and sarcomeres (Joumaa et al., 2008; Leonard et al., 2010). Residual force enhancement is known to increase with the magnitude of stretch (Abbott and Aubert, 1952; Edman et al., 1978), and is long lasting (more than 20 s in cat soleus), but can be abolished instantaneously by deactivating the muscle long enough for force to drop to zero (Morgan et al., 2000; Herzog and Leonard, 2002). Furthermore, it has been found that the energy cost per unit of force is reduced by more than 15% in the force enhanced compared to the purely isometric reference state, while stiffness was the same for the two conditions, suggesting that the proportion of attached cross-bridges was similar for the force enhanced and the isometric reference states (Joumaa and Herzog, 2013). This finding suggests that residual force enhancement is accompanied by an increase in muscle efficiency.

Knowing that residual force enhancement may contribute to the increase in force following SSCs, and that residual force enhancement is associated with an increase in muscle efficiency, it is plausible that efficiency is also increased following SSCs. SSCs are common during everyday movement and an increase in muscle efficiency caused by SSCs might be more important than the mechanical advantage of an increase in force.

The purpose of this study was to investigate the energy cost of force production at steady-state following SSCs and compare it to the energy cost following pure shortening contractions. We hypothesized that the energy cost is reduced following SSCs compared to pure shortening contractions. Skinned fibers from rabbit psoas were used for all experiments, and energy cost (ATP consumed) was quantified by measuring the amount of phosphate released during contraction using a malachite green assay (Fujita et al., 1999; Karpowich et al., 2015; Utter et al., 2015). Fibers were stretched to a long sarcomere length of 3.2 μm before being shortened (Fukutani et al., 2017) in order to maximize residual force enhancement and its potential effect on force production.

## Materials and Methods

### Skinned Fiber Preparation

Rabbits were euthanized by an intravenous injection of 1 ml of a pentobarbital solution (240 mg/ml), a protocol approved by the University of Calgary’s Animal Care and Ethics Committee. Strips of psoas muscle were dissected, tied to small wooden sticks and stored in a rigor solution for 12 h at 4°C, then in a rigor-glycerol (50:50) solution at −20°C for 2 weeks (Joumaa and Herzog, 2013). On the day of the experiments, a single fiber segment was dissected from the skinned muscle biopsy using a binocular microscope and transferred to an experimental glass chamber containing the relaxing solution. One end of the fiber was glued to the hook of a length controller and the other end to the hook of a force transducer (Aurora Scientific Inc, Ontario, Canada), allowing control of length and force measurements, respectively. Sarcomere lengths were measured using optical diffraction of a He-Ne laser beam. All experiments were performed at ∼24°C.

### Mechanical Tests

#### SSC and Active Shortening to a Sarcomere Length of 2.6 μm

**FIGURE 1:**
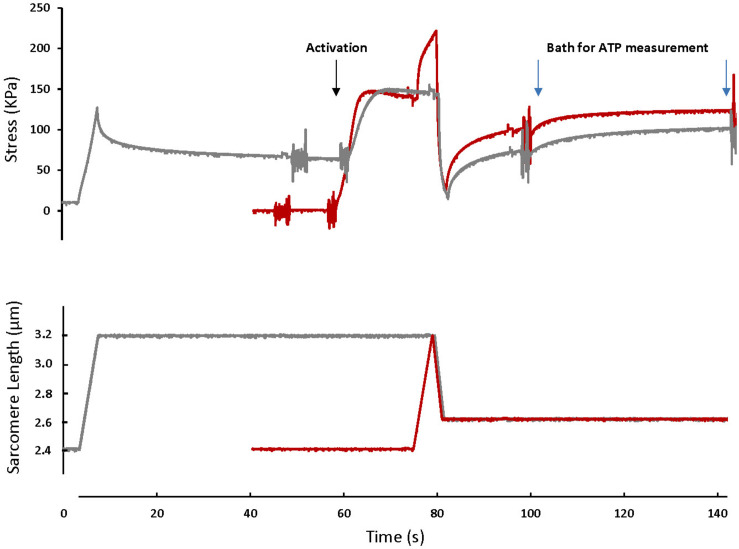
A typical fiber response to an active shortening without a prior stretch (gray) and a stretch-shortening cycle (red). For the active shortening contraction, the fiber was passively stretched from a SL of 2.4 μm to a SL of 3.2 μm, activated and then actively shortened to a SL of 2.6 μm. After 15 s, the fiber was transferred to a fresh activating solution for 40 s and then deactivated. For the SSC contraction, the fiber was activated at a SL of 2.4 μm, actively stretched to a SL of 3.2 μm and immediately shortened to a SL of 2.6 μm. After 15 s the fiber was transferred to a bath of fresh activating solution for 40 s. The activating solutions in which the fibers were bathed for 40 s were used for ATP measurement. Note that the rate of force redevelopment and the steady-state force after shortening are smaller in the active shortening without prior stretch test compared to the SSC test. A fiber was activated by adding first a washing solution (free of EGTA and calcium) and then an activating solution (with high concentration of calcium). The noise on the graph indicates the time when the solution was changed. Active shortening lasted long enough to favor force depression.

For the SSC test (SSC-2.6), skinned fibers (*n* = 12) were set at an average sarcomere length (SL) of 2.4 μm (L_0_) in a relaxing solution, activated, stretched to a SL of 3.2 μm in 4 s, and immediately shortened to a SL of 2.6 μm in 2 s. Fibers were held at this length for 15 s, and then transferred to another bath of activating solution for 40 s, then to a relaxing solution (Figure 1). For the active shortening contractions without a prior stretch (CTL-2.6), the same fibers were passively stretched to an average SL of 3.2 μm in 4 s, held for 40 s, and then activated and shortened to a SL of 2.6 μm in 2 s. Fibers were held at this length for 15 s, and then transferred to another bath of activating solution for 40 s, then to a relaxing solution (Figure 1).

Fibers were given a 5 min rest period between tests. After each test, the activating solutions in which the fiber was bathed for 40 s were collected for the assessment of ATP consumed.

#### SSC and Active Shortening to a Sarcomere Length of 3.0 μm

Experiments were identical to those outlined in the previous paragraph, except that the active shortening performed during the SSC (SSC-3.0) and shortening (CTL-3.0) contractions was from a SL of 3.2 μm to a SL of 3.0 μm in 1 s.

### Metabolic Cost

Metabolic cost was quantified by measuring ATPase activity using the malachite green phosphate method (Kodama et al., 1986; Fujita et al., 1999; Karpowich et al., 2015; Utter et al., 2015). The activating solution in which the fiber was bathed during the last 40 s of activation following shortening in the SSC-2.6, SSC-3.0, CTL-2.6, and CTL-3.0 tests was diluted 10 times. Then, inorganic phosphate (Pi) was determined by measuring the absorbance at 620 nm of the green complex formed between malachite green, molybdate and Pi (Sigma-Aldrich MAK307).

**FIGURE 2:**
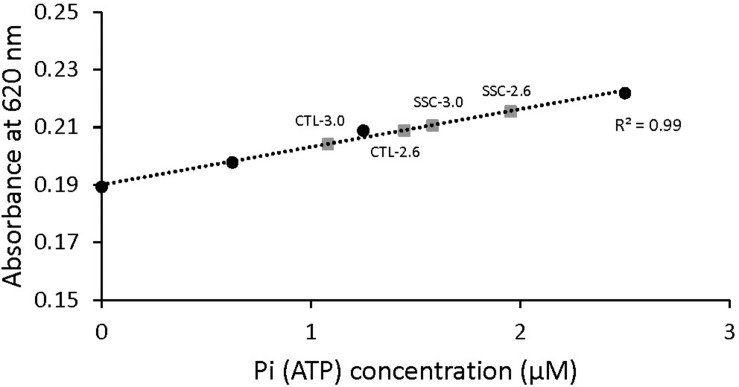
Malachite green absorbance as a function of Pi (ATP) concentration. The standard curve was established by adding known amounts of Pi. When the concentration of Pi increased, the amount of malachite green increased and therefore the absorbance was increased too. Examples of the absorbance of malachite green in the diluted solutions of active shortening (CTL-2.6 and CTL-3.0) and SSCs (SSC-2.6 and SSC-3.0) contractions are also shown. The malachite green assay used in this study could detect differences in the concentration of Pi in the order of 0.1 μM.

The absorbance signal was calibrated by using a known amount of Pi and monitoring the green complex absorbance. Sensitivity of this method to small changes in ATP concentration was also tested (Figure 2). Pi produced by the fibers during activation was calculated and converted to amount of ATP used during contraction by assuming that ATP used during contraction is equal to Pi produced (Huxley, 1957).

### Stiffness Measurements

An additional group of 12 fibers was used to measure stiffness after the stretch shortening cycles and purely active shortening stretches. Stiffness was measured using sinusoidal length changes of a peak-to-peak amplitude of 0.2% of L_0_ at 0.5 kHz (Regnier et al., 1998) for 50 ms at sarcomere lengths of 2.6 and 3.0 μm, 15 s following shortening in the SSC and active shortening tests.

### Metabolic Cost After Residual Force Enhancement

It has been shown that residual force enhancement is accompanied by a decrease in the energy cost per unit of force (Joumaa and Herzog, 2013). In order to check our ability to reproduce similar results in this study, we used another group of fibers (*n* = 5) to determine the energy cost of force production after active stretch compared to a purely isometric reference contraction performed at the same final fiber length. For the reference contraction condition, fibers were set at an average sarcomere length of 3.2 μm, activated, held for 15 s, transferred to another activating solution bath for 40 s and then deactivated. After a rest period of 5 min, the active stretch contraction was performed. Fibers were activated at a sarcomere length of 2.4 μm, then actively stretched to a sarcomere length of 3.2 μm. Fibers were held at this length for 15 s, transferred to another bath of activating solution for 40 s, and then relaxed. After a rest period of 5 min, reference and active stretch contraction tests were repeated. The activating solutions in which the fibers were bathed for 40 s were used for metabolic cost assessment.

### Data Analysis

#### Steady-State Stress

Steady-state total stress at sarcomere lengths of 2.6 and 3.0 μm was determined as the average stress produced during the last 40 s of activation following shortening in the SSC-2.6, SSC-3.0, CTL-2.6, and CTL-3.0 tests.

#### Mechanical Work

Mechanical work during shortening in the SSC and active shortening tests was calculated by trapezoidal numerical integration of the total force-displacement, curve during the shortening phase.

#### Metabolic Cost

The amount of ATP used during the last 40 s of activation following shortening in the SSC-2.6, SSC-3.0, CTL-2.6, and CTL-3.0 tests was divided by the average steady-state total force produced during this time in order to obtain the ATPase activity per unit of force.

#### Stiffness

Stiffness, expressed as Young’s modulus (N/mm^2^), was measured from the sinusoidal length changes applied to the fiber, as the average peak-to-peak change in stress divided by the peak-to-peak change in length for 10 consecutive cycles.

### Statistical Analysis

The outcome measures were compared between the SSC condition and the pure isometric shortening condition at a given sarcomere length (at a sarcomere length of 2.6 μm: SSC-2.6 vs. CTL-2.6, and at a sarcomere length of 3.0 μm: SSC-3.0 vs. CTL-3.0), and therefore the non-parametric Wilcoxon matched-pair signed-rank test (α = 0.05) was used. Bonferroni correction was used for multiple comparisons.

## Results

Figure 1 shows the force-time history of a typical SSC and active shortening experiment.

**FIGURE 3:**
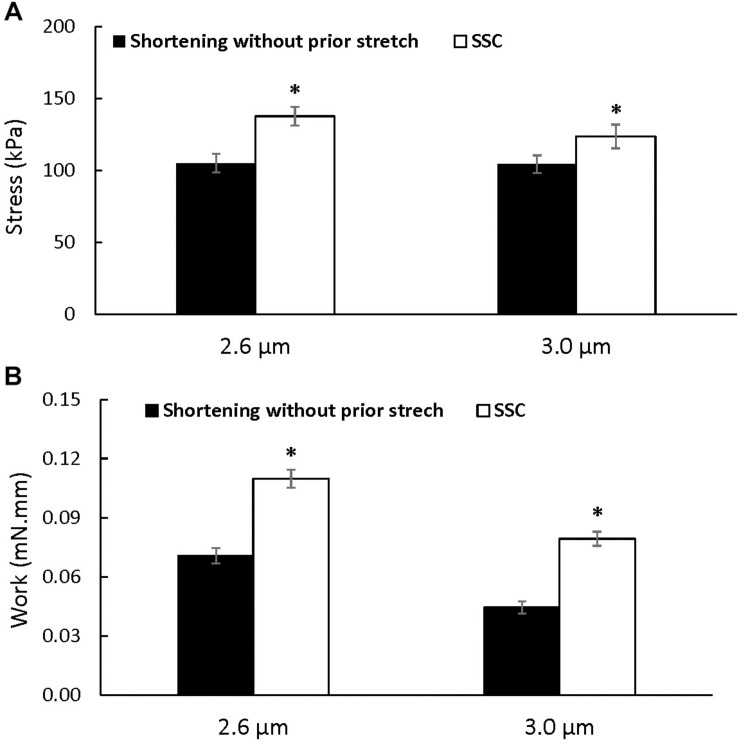
Average total stress **(A)** and work performed during shortening **(B)** (±SEM) for the active shortening and SSC contractions at sarcomere lengths of 2.6 and 3.0 μm. Total stress and the work performed during shortening were greater after SSCs compared to the pure shortening contractions. ^∗^Indicates a significant difference from the corresponding pure active shortening contraction.

*Steady-state force*: Steady-state total force produced during the last 40 s of activation was greater after the SSCs compared to the pure shortening conditions at sarcomere lengths of 2.6 μm (*p* = 0.009) and 3.0 μm (*p* = 0.003) (Figure 3A). On average, the increase in total force following the SSCs was (mean ± SEM) 29.8 ± 3.2% and 17.9 ± 1.8% at sarcomere lengths of 2.6 and 3.0 μm, respectively.

### Mechanical Work

Mechanical work during the shortening phase was enhanced in the SSC conditions compared to the pure shortening conditions (*p* = 0.002 and 0.002 for sarcomere lengths of 2.6 and 3.0 μm, respectively) (Figure 3B).

### Metabolic Cost

**FIGURE 4:**
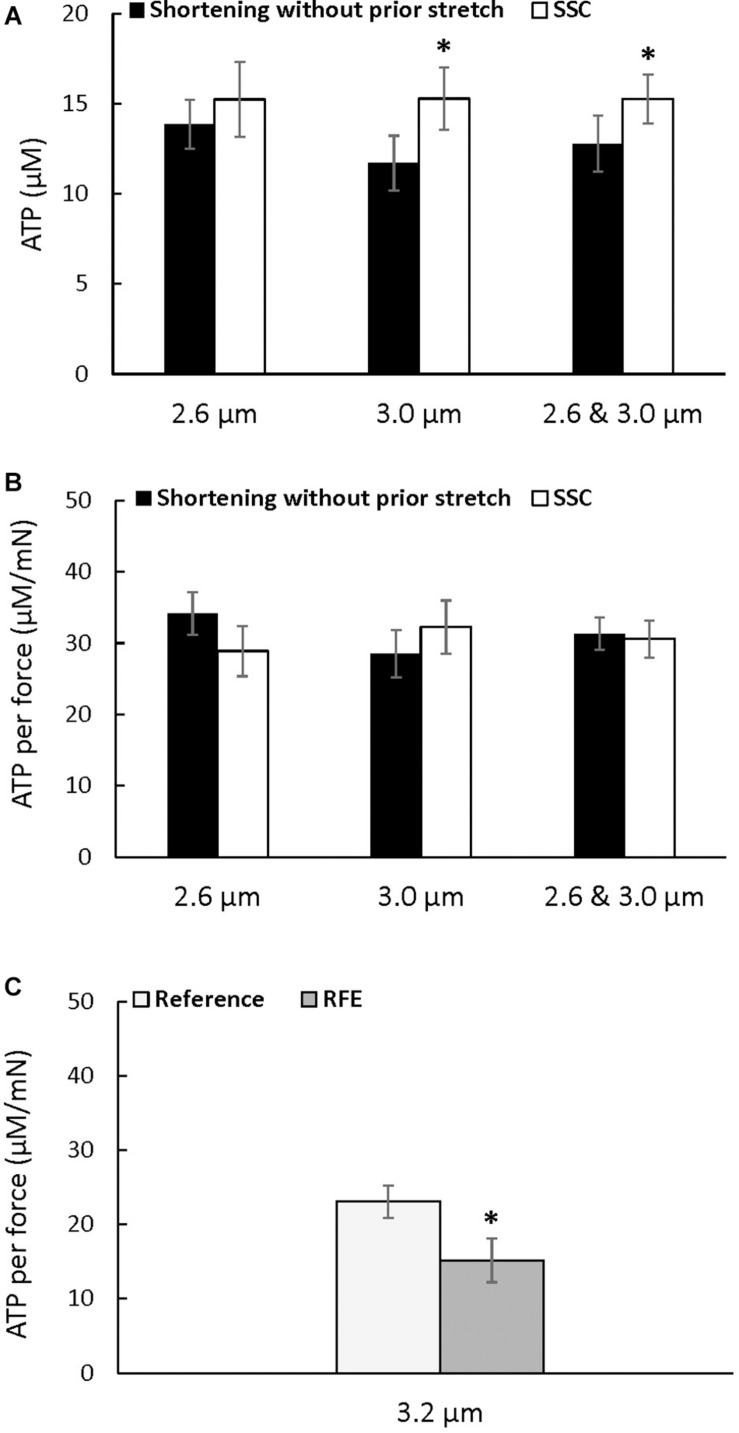
Average ATP consumption **(A)**, average ATP consumption per unit of force **(B)** for the active shortening and the SSC contractions at sarcomere lengths of 2.6, 3.0, and 2.6 μm and 3.0 μm combined, and average ATP consumption per unit of force for the residual force enhancement **(C)** (±SEM). ATP consumption was greater after SSCs at a sarcomere length of 3.0 μm, and when 2.6 μm and 3.0 μm were combined, compared to the pure shortening contractions. ATP consumption per unit of force was not different after SSCs compared to the corresponding active shortening contractions but was reduced in the RFE compared to the isometric reference contractions. The residual force enhancement tests were performed twice, and results were combined for the first and second reference and active stretch contractions. ^∗^Indicates a significant difference from the corresponding pure shortening contraction (*p* < 0.05).

There was a trend for an increase in the absolute amount of ATP consumed at a sarcomere length of 2.6 μm after the SSC condition compared to the pure shortening contraction (*p* = 0.083), but the absolute amount of ATP consumed was higher after the SSC contraction compared to the pure shortening condition at a sarcomere length of 3.0 μm (*p* = 0.016), and when the data for both sarcomere lengths was combined (*p* = 0.022) (Figure 4A). However, ATP consumption per unit of total force was not different between the SSC and the pure shortening conditions at sarcomere lengths of 2.6 μm (*p* = 0.221) and 3.0 μm (*p* = 0.262) (Figure 4B).

### Stiffness

**FIGURE 5:**
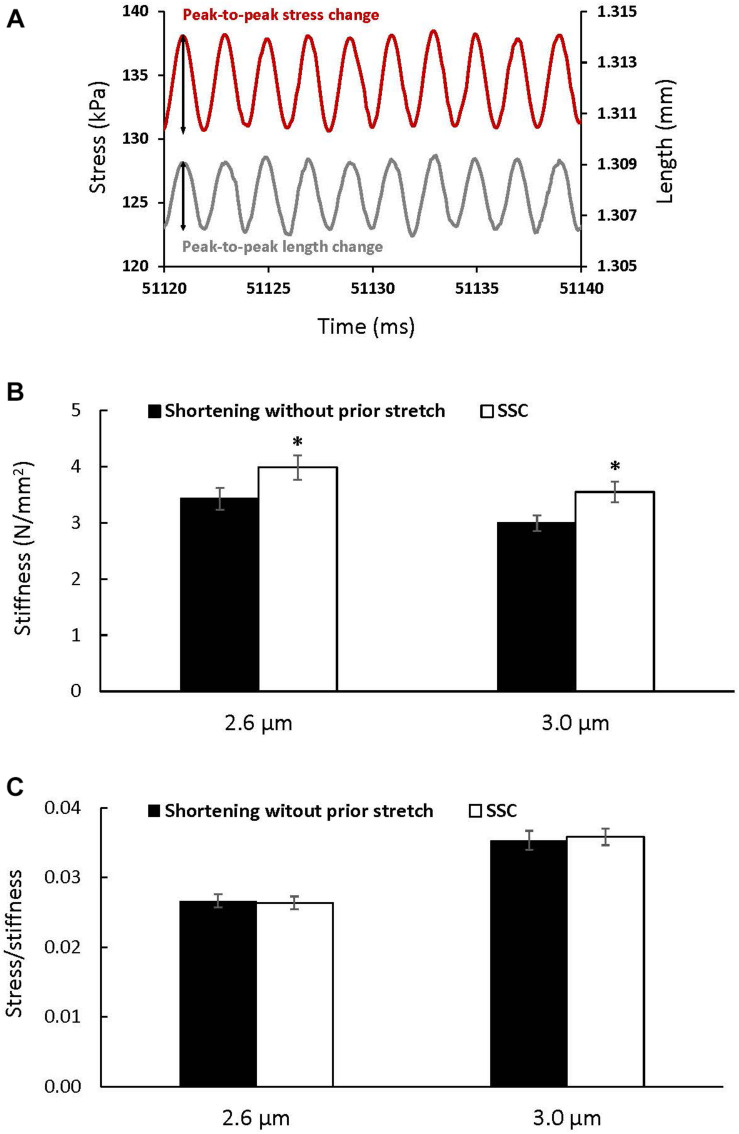
The change in stress (red) in response to the sinusoidal length changes (gray) performed to measure stiffness **(A)**, and average stiffness **(B)** and stress relative to stiffness **(C)** (±SEM) for the active shortening and SSC contractions at sarcomere lengths of 2.6 and 3.0 μm. Stiffness was calculated as the average change in stress (peak-to-peak stress change) divided by the change in the initial fiber length (peak-to-peak length change expressed as strain) for 10 consecutive cycles. ^∗^Indicates a significant difference from the corresponding active shortening contraction (*p* < 0.05).

Stiffness (Figure 5A) increased after the SSC conditions compared to the pure shortening conditions at sarcomere lengths of 2.6 μm (*p* = 0.002) and 3.0 (*p* = 0.002) by (mean ± SEM) 16 ± 1 and 18 ± 2%, respectively (Figure 5B). Total stress observed at steady-state after SSCs and active shortening tests normalized to stiffness was not different between the SSC and the pure shortening conditions at sarcomere lengths of 2.6 μm (*p* = 0.687) and 3.0 μm (*p* = 0.227) (Figure 5C).

### Metabolic Cost of Residual Force Enhancement

The energy cost per unit of total force was significantly reduced after active stretch compared to the purely isometric reference contraction (*p* = 0.022) (Figure 4C), confirming the previously reported reduction in the energy cost of total force production after an active stretch (Joumaa and Herzog, 2013).

## Discussion

The purpose of this study was to investigate the energy cost of force production at steady-state after a SSC and compare it to the energy cost of force after shortening contractions without a prior stretch. Our main finding was that force and ATP consumption were enhanced at steady-state after SSCs, and that the energy cost per unit of force was the same for both experimental conditions.

Active stretch and residual force enhancement have been associated with a substantial increase in muscle efficiency, as measured by a reduction in the metabolic energy cost per unit of force (Joumaa and Herzog, 2013). Based on these earlier findings, we hypothesized that the metabolic energy cost per unit of force after SSCs may also be reduced compared to pure shortening contractions. We used long stretches (to a sarcomere length of 3.2 μm) in our experimental protocols in order to maximize the effect of active stretching on force in the SSCs. However, the steady-state force was enhanced following SSCs compared to the corresponding pure shortening contractions and so was the ATP consumption, resulting in similar metabolic costs per unit of force for the two experimental conditions, and thus, a similar efficiency.

The absence of the expected increase in muscle efficiency following SSCs may be due to the fact that the increase in force following the SSCs was likely accompanied by a corresponding increase in the proportion of attached cross-bridges compared to the pure shortening contractions. This suggestion is supported by the proportional increase in stiffness with force and the absence of a change in the ratio of force to stiffness in the SSC compared to the corresponding pure shortening conditions (Figures 5B,C). Stiffness measurements must be viewed with caution when interpreting them as a measure of the proportion of attached cross-bridges alone, since other elements like titin contribute to active stiffness, and this titin-based stiffness increases after activation (Powers et al., 2020) and stretch (Herzog, 2014). Although titin contribution to passive stiffness is minimal compared to that of cross-bridges (about 0.04 pN/nm per titin molecule (Linke et al., 1998) vs. 1.21 pN/nm per cross-bridge (Linari et al., 2007), Powers et al. (2020) have shown that titin-associated stiffness during muscle contraction is about two order of magnitudes larger than what has been reported for passive conditions. This suggests that in our experiments titin contributes to stiffness in addition to the cross-bridges. Furthermore, knowing that the force produced by titin does not require ATP, the fact that there is a trend toward an increase in absolute ATP consumption after SSC-2.6 and not a statistically significant increase in ATP consumption, suggests that the observed increase in stiffness following SSC-2.6 was in part caused by an increase in titin force after active stretch and shortening, in addition to the increase in the proportion of attached cross-bridges. Since the increase in stiffness after SSC-3.0 was accompanied by an increase in absolute ATP consumption, the contribution of titin to the increase in stiffness after SSC-3.0 seems lower than at SSC-2.6. Although titin-based force is normally lower at a sarcomere length of 2.6 μm compared to a sarcomere length of 3.0 μm, titin contribution to force and stiffness at steady-state after SSC-2.6 seems greater than that after SSC-3.0. We speculate that this could result from a greater restoring force developed by a stretch-stiffened titin when actively shortened to a shorter sarcomere length of 2.6 μm compared to a sarcomere length of 3.0 μm.

Why do the proportion of attached cross-bridges and cross-bridge-based stiffness increase at steady-state after SSCs compared to the corresponding pure shortening contractions? It has been observed that the steady-state isometric force after active shortening of muscles is reduced compared to the purely isometric force at the corresponding length (Abbott and Aubert, 1952; Edman, 1975; Granzier and Pollack, 1989). Furthermore, experiments using whole muscles (Lee and Herzog, 2003) and single fibers (Sugi and Tsuchiya, 1988; Joumaa et al., 2012; Pinnell et al., 2019) have consistently shown that force depression is associated with a proportional decrease in stiffness that has been interpreted as a decrease in the proportion of attached cross-bridges, compared to isometric contractions at the corresponding length. Our finding that the proportion of attached cross-bridges after SSCs was greater than that after a shortening contraction suggests that, somehow, an active stretch preceding a shortening contraction effectively prevented the reduction in cross-bridges during a pure shortening contraction.

It is hard to reconcile the suggested increase in attached cross-bridges observed after SSCs compared to pure shortening contractions with the stress-induced inhibition of cross-bridge hypothesis suggested by Marechal and Plaghki (1979) to explain force depression. Marechal and Plaghki (1979) suggested that active shortening might be associated with a stress-induced inhibition of cross-bridge formation in the newly formed overlap zone created during the active shortening. This hypothesis has been supported by data showing that the decrease in force at steady-state after an active shortening increases with the amount of stress and mechanical work performed during shortening: the greater the work during shortening, the lower the stiffness and the lower the force at steady-state after shortening, likely because of a greater inhibition of cross-bridge formation (Meijer et al., 1997; Herzog et al., 2000; Joumaa et al., 2017). However, although the mechanical work performed during the shortening phase of the SSC was greater than the work performed during the pure shortening contractions (Figure 3B), stiffness and force were greater after SSCs compared to the corresponding shortening contractions. Similar findings, showing great force following SSCs despite a high amount of work produced during the shortening phase, were obtained in previous studies using fibers (Fukutani et al., 2017; Fukutani and Herzog, 2019; Fukutani and Isaka, 2019) and *in vivo* muscles (Fortuna et al., 2018, 2019; Hahn and Riedel, 2018). One possible explanation for these findings may be that the stress-induced inhibition of cross-bridges during the shortening phase of the SSC was compensated for by an increase in cross-bridge recruitment and formation during and after the initial lengthening phase of the SSC. However, if this interpretation was correct, residual force enhancement at steady-state following active stretching would be accompanied by an increase in the proportion of attached cross-bridges compared to the corresponding pure isometric contraction. However, this is not the case and the proportion of attached cross-bridges does not increase in the force enhanced state compared to the corresponding pure isometric contractions (Sugi and Tsuchiya, 1988; Joumaa and Herzog, 2013). Therefore, an increase in cross-bridge recruitment would not occur in the stretch phase of the SSCs but would have to occur in the shortening phase. A plausible explanation for a reduced inhibition of cross-bridge formation during the shortening phase of SSC was suggested by Fortuna et al. (2019) and further supported by Tomalka et al. (2020). These authors argued that in pure shortening contractions, work is produced primarily by cross-bridges, while in SSC contractions, a substantial amount of the shortening work is produced by the recoil of the passive structures that elongated during the stretch phase of the SSCs (Fortuna et al., 2019). In this case, the stress on actin filaments, and associated cross-bridge inhibition, might be reduced compared to pure shortening contractions, which in turn, could result in the observed increase in the proportion of attached cross-bridges following SSCs compared to pure shortening contractions.

Another possibility is based on the direct effect of the recruitment of the passive structures (titin) during the active stretch on the contractile filaments. It has been demonstrated using myofibrillar models (Hanft et al., 2013) and x-ray diffraction (Irving et al., 2011; Ait-Mou et al., 2016) that titin passive force induces stretch-based alterations in the structural arrangement and flexibility of the contractile filaments. Therefore, we speculate that the stretch phase of the SSCs and the associated substantial increase in titin-based force could stabilize the contractile filaments and reduce actin conformational distortion and stress-induced inhibition of cross-bridges during the shortening phase. This will result in more cross-bridges at steady-state following SSCs compared to pure shortening contractions, in which titin-based force is relatively low and has limited capacity to stabilize the nanostructure of the contractile filaments. Nevertheless, further research is warranted to investigate the impact of active lengthening on titin, the contractile filaments and cross-bridge formation and inhibition during SSCs.

## Limitations and Future Directions

In this study, we investigated whether the increase in force after a SSC was accompanied by an increase in muscle efficiency, and found that muscle efficiency at steady-state after a SSC was not improved. This result might not apply readily to everyday activities as most daily movements involving SSCs are not followed by long isometric contractions. However, this result provides insights into the potential mechanisms of the increase in force at steady-state after SSCs. A major limitation of this study was that ATP consumption could not be measured in real time during the dynamic phases of SSCs. Future experiments should aim at addressing this limitation by assessing the energy cost of force in real time during the stretching and shortening phases of SSCs. Furthermore, although the frequency of the sinusoidal perturbations used in this study to measure stiffness has been used by others (e.g., Regnier et al., 1998), it could be argued that a greater frequency should have been used to prevent the effect of force recovery on stiffness.

## Conclusion

We have shown that force was enhanced at steady-state following SSCs, but there was no difference in the metabolic energy cost of force following SSCs and pure shortening contractions. The increase in force is likely caused by an increase in the proportion of attached cross-bridges and titin stiffness following SSCs compared to pure shortening contractions.

## Data Availability Statement

The raw data supporting the conclusions of this article will be made available by the authors, without undue reservation.

## Ethics Statement

The animal study was reviewed and approved by the University of Calgary’s Animal Care and Ethics Committee.

## Author Contributions

VJ, AF, and WH conceived and designed research, interpreted results of experiments, edited and revised manuscript, and approved final version of manuscript. VJ performed the experiments, analyzed the data, and drafted the manuscript. All authors contributed to the article and approved the submitted version.

## Conflict of Interest

The authors declare that the research was conducted in the absence of any commercial or financial relationships that could be construed as a potential conflict of interest.
